# Efficient In Vitro and In Vivo Anti-Inflammatory Activity of a Diamine-PEGylated Oleanolic Acid Derivative

**DOI:** 10.3390/ijms22158158

**Published:** 2021-07-29

**Authors:** Fatin Jannus, Marta Medina-O’Donnell, Veronika E. Neubrand, Milagros Marín, Maria J. Saez-Lara, M. Rosario Sepulveda, Eva E. Rufino-Palomares, Antonio Martinez, Jose A. Lupiañez, Andres Parra, Francisco Rivas, Fernando J. Reyes-Zurita

**Affiliations:** 1 Department of Biochemistry and Molecular Biology I, Faculty of Sciences, University of Granada, Av. Fuentenueva, 18071 Granada, Spain; fatin@correo.ugr.es (F.J.); mmarin@ugr.es (M.M.); mjsaez@ugr.es (M.J.S.-L.); evaevae@ugr.es (E.E.R.-P.); jlcara@ugr.es (J.A.L.); 2 Department of Organic Chemistry, Faculty of Sciences, University of Granada, Av. Fuentenueva, 18071 Granada, Spain; aramon@ugr.es (A.M.); aparra@ugr.es (A.P.); 3 Department of Cell Biology, Faculty of Sciences, University of Granada, Av. Fuentenueva, 18071 Granada, Spain; neubrand@ugr.es (V.E.N.); mrsepulveda@ugr.es (M.R.S.)

**Keywords:** oleanolic acid, triterpenes derivatives, diamine-(PEG)ylated oleanolic acid, OADP, anti-inflammatory mechanism, RAW 264.7 cell line, TPA-induced acute ear edema

## Abstract

Recent evidence has shown that inflammation can contribute to all tumorigenic states. We have investigated the anti-inflammatory effects of a diamine-PEGylated derivative of oleanolic acid (OADP), in vitro and in vivo with inflammation models. In addition, we have determined the sub-cytotoxic concentrations for anti-inflammatory assays of OADP in RAW 264.7 cells. The inflammatory process began with incubation with lipopolysaccharide (LPS). Nitric oxide production levels were also determined, exceeding 75% inhibition of NO for a concentration of 1 µg/mL of OADP. Cell-cycle analysis showed a reversal of the arrest in the G0/G1 phase in LPS-stimulated RAW 264.7 cells. Furthermore, through Western blot analysis, we have determined the probable molecular mechanism activated by OADP; the inhibition of the expression of cytokines such as TNF-α, IL-1β, iNOS, and COX-2; and the blocking of p-IκBα production in LPS-stimulated RAW 264.7 cells. Finally, we have analyzed the anti-inflammatory action of OADP in a mouse acute ear edema, in male BL/6J mice treated with OADP and tetradecanoyl phorbol acetate (TPA). Treatment with OADP induced greater suppression of edema and decreased the ear thickness 14% more than diclofenac. The development of new derivatives such as OADP with powerful anti-inflammatory effects could represent an effective therapeutic strategy against inflammation and tumorigenic processes.

## 1. Introduction

Inflammation is an innate natural process of the immune system. When persisting for a long period, this process can trigger various chronic diseases such as autoimmune disorders, arthritis, cardiovascular diseases, diabetes, Parkinson’s, and cancer. In recent years, new natural compounds with anti-inflammatory properties have been identified and studied, offering the potential to decrease excessive inflammation associated with many diseases [1]. Our research group has recently investigated the anti-inflammatory effect of various diclofenac derivatives [2].

Previous studies have reported the in vitro and in vivo anti-inflammatory effects of various triterpenoids. Several triterpenic saponins have significantly reduced NO production in LPS-stimulated RAW 264.7 cells, inhibiting the release of pro-inflammatory cytokines, such as the tumor necrosis factor (TNF-α), interleukine-1 β (IL-1β), interleukine-6 (IL-6), and interleukine-8 (IL-8) [3]. In addition, SH479, a derivative of betulinic acid, ameliorated experimental autoimmune encephalomyelitis in a mouse model by modulating the Th17/Treg balance, on inhibiting the signal transducer and activator of transcription 3 (STAT3) and the nuclear factor kappa-light-chain-enhancer of activated B-cell (NF-κB) pathways and activating the STAT5 pathway [4]. Furthermore, oleanolic acid regulated the production of anti-inflammatory cytokines and decreased the production of pro-inflammatory cytokines in mice with experimental autoimmune myocarditis [5]. Similarly, in a mouse model of experimental autoimmune encephalomyelitis, oleanolic acid acetate suppressed the production of pro-inflammatory cytokines IL-1β, IL-6, INF-γ, and TNF-α by regulating toll-like receptor 2 (TLR2) signaling [6]. A synthetic derivative of oleanolic acid (CDDO-Im) inhibited IL-6 and IL-17 expression and palliated dextran sulfate sodium (DSS) induced colitis in mice, as well as inhibited STAT3 activation [7]. Additional studies demonstrated that acetylated and methylated derivatives of oleanolic acid elicited a better anti-inflammatory response in male Wistar rat used as models of inflammation than did triterpenic acid [8,9].

Olives are a rich source of pentacyclic triterpenes such as oleanolic and maslinic acids. Oleanolic acid is present in high concentrations in olive pomace, which represents 37% of the total triterpenoids in this fruit [10]. The concentration of this compound in extra virgin olive oil is 57.3 mg/kg, whereas in pomace oil is 5592 mg/kg. The greatest increase in triterpenes as the quality of the oil decreases is because these compounds are found mainly in the outer waxy layer of the olive fruit so that successive pressing and extractions favor their elimination [11]. In this study, oleanolic acid was obtained from solid olive oil wastes, using the method described in [12].

PEGylation is a technique that enables ethylene glycol units to bond to drugs, forming linear or branched polymers with different molar masses, which improve their bio-distribution and bioavailability [13]. Our research group performed PEGylation reactions, covalently linking a polyethylene glycol (PEG) reagent to oleanolic acid (OA) and thereby producing a series of derivatives with greater solubility in aqueous media [14,15,16]. One of these PEGylated derivatives of oleanolic acid (OADP) has been studied due to its high cytotoxicity in Hep G2 cancer cells [17]. In the present study, we found that the cytotoxicity of OADP in human embryo WRL68 non-tumor liver cells was 39-fold lower than in the Hep G2 cancer cell line. Therefore, we studied the potential anti-inflammatory effect of OADP, since inflammation is related to cancer and plays a key role in tumorigenesis [18]. In this sense, an understanding of the molecular mechanisms involved in the inflammatory process can help to prevent certain chronic diseases and even some types of cancer.

In the inflammation process, different proteins such as protein kinases, PI3K/AKT, JAK, and MAPK are activated [19], so transcription factors such as STAT, AP-1 or NF-κB are activated, inducing the expression of pro-inflammatory proteins such as COX-2, iNOS, TNF-α, IL-1β, and IL-6 [20,21]. Of these, COX-2 and iNOS produce the intermediates of the inflammation process, PGE-2 and nitric oxide (NO), respectively. In addition, NO can regulate the acute and chronic inflammatory process. COX-2 is activated in immune cells such as macrophages, producing PGs that induce different inflammatory states [22,23]. Disruption of the activation of this process can lead to aberrant cell growth, cancer cell transformation, angiogenesis, and metastasis. To evaluate the in vivo topical anti-inflammatory effects of OADP, we used the animal model of induced ear edema in mice [24]. In this model, inflammation is provoked by topical treatment with 12-O-tetradecanoylphorbol 13-acetate (TPA), a phlogistic factor that causes the activation of MAP kinase proteins, activating the molecular pathway for the onset of the inflammatory process [25].

In this work, we examine the anti-inflammatory effect of OADP in LPS-stimulated RAW 264.7 cells and in mice having acute TPA-induced ear edema. Firstly, we conducted in vitro studies, determining NO release at different sub-cytotoxic concentrations of OADP after 72 h of incubation. The inhibition of NO production was accompanied by a reversal of G0/G1 phase arrest in LPS-stimulated RAW 264.7 cells. Secondly, we studied the underlying anti-inflammatory molecular mechanisms activated by OADP. Thus, OADP inhibited the expression of the main inflammatory cytokines such as TNF-α, IL-1β, iNOS, COX-2, and a regulated protein such as p-IκBα. Furthermore, we evaluated the anti-inflammatory effect in mice with acute ear edema by using a series of morphological measurements, histopathological analyses, and IL-6 induction levels.

## 2. Results

### 2.1. Cytotoxicity of OADP in the Proliferation of RAW 264.7 Cells

**Figure 1:**
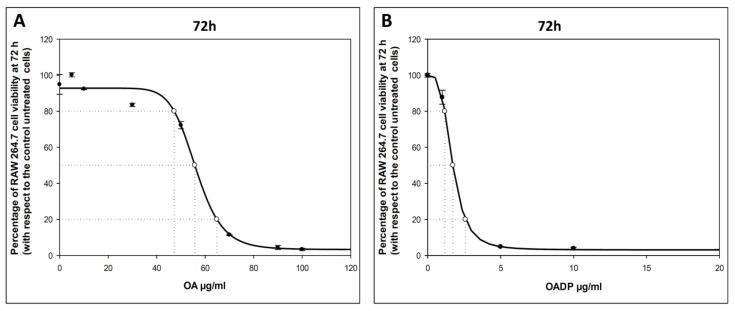
Effect of OA (**A**) and OADP (**B**) on cell proliferation of RAW 264.7 macrophage murine cells, after treatment with the compounds for 72 h. Each point represents the mean value ± S.D. of at least two independent experiments performed in triplicate.

To evaluate the cytotoxic effects of OADP and OA on RAW 264.7 murine macrophage cells, we incubated these cells at increasing concentrations (0–100 µg/mL) of OADP for 72 h (Figure 1). Cell viability was analyzed using the MTT assay, where the tetrazolium dye was transformed into formazan in the mitochondria of viable cells, its absorbance measured at 570 nm. The OADP concentration required for 50% cell growth inhibition (IC_50_) after 72 h of incubation was less than 2 µg/mL (IC_50_ = 1.72 ± 0.10 µg/mL), and was 56 µg/mL for OA. These cell viabilities with these compounds were determined to calculate the sub-cytotoxic concentrations at which to perform the anti-inflammatory tests. ¾ IC_50_ = 1.29 µg/mL, ½ IC_50_ = 0.86 µg/mL, and ¼ IC_50_ = 0.43 µg/mL for OADP. ¾ IC_50_ = 41.78 µg/mL, ½ IC_50_ = 27.86 µg/mL, and ¼ IC_50_ = 13.92 µg/mL for OA. These data for diclofenac were obtained from Galisteo et al. 2020 [2]. The use of these sub-cytotoxic concentrations ensures that the anti-inflammatory activity is due to an inflammatory process and not to its cytotoxicity.

### 2.2. Nitric Oxide Production

In the inflammation process, NO is produced by inducible nitric oxide synthase (iNOS) and, as the main pro-inflammatory mediator, plays a key role in the immune system. Murine macrophage cells RAW 264.7 were used to study the anti-inflammatory effect of OADP, which induces a significant release of NO during the inflammatory process. Therefore, the anti-inflammatory activity of OADP was assessed with the Griess method by measuring the nitrite concentration, proportional to the NO released, in the cell culture medium. These macrophages (RAW 264.7) were activated with LPS for 24 h after the addition of OADP. The following sub-cytotoxic concentrations were used: ¾ IC_50_, ½ IC_50_, and ¼ IC_50_.

**Figure 2:**
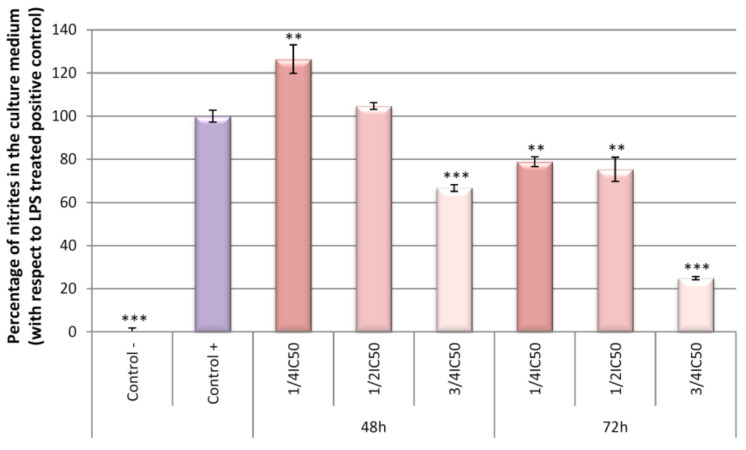
Effect of OADP on NO production in RAW 264.7 macrophage murine cells, after treatment with OADP for 48 h and 72 h at the ½ IC_50_ and ¾ IC_50_ concentrations. Data represent the mean ± S.D. of at least two independent experiments performed in triplicate. Key: (**) *p* < 0.01 and (***) *p* < 0.001, with respect to LPS-treated control cells (positive control).

**Figure 3:**
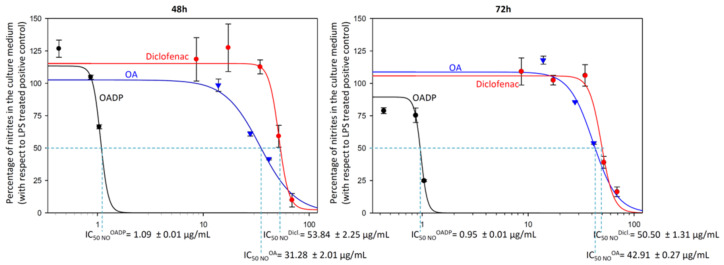
Sigmoidal curves of the effect of OADP (black), OA (blue) and diclofenac (red) on nitrite release in RAW 264.7 cells, after treatment with the compounds for 48 h and 72 h. Data represent the mean ± S.D. of at least two independent experiments performed in triplicate.

NO production at 24, 48, and 72 h of incubation (Figure 2) was hardly detectable in the negative control (untreated cells), compared to the positive control (cells treated only with LPS). After 24 h of incubation, OADP did not cause any anti-inflammatory effect. Meanwhile, at 48 h of incubation, a slight anti-inflammatory effect was noted at the ¾ IC_50_ concentration, with a 33% inhibition of NO production. However, 72 h of incubation gave rise to a strong anti-inflammatory effect at the ¾ IC_50_ concentration, with 75% inhibition of NO production. In addition, slight anti-inflammatory effects were detected at the ½ IC_50_ and ¼ IC_50_ concentrations, with approximately 25% inhibition of NO production, with respect to the increase between the positive and negative control (Figure 2). At the concentrations tested, OA and diclofenac exerted no inhibitory effect on NO production in LPS-activated RAW 264.7 cells (Figure 3).

To compile more data on the anti-inflammatory effect of OADP, we calculated the doses that result in 50% NO inhibition (IC_50 NO_) for OADP and compared them with those of OA and diclofenac, in each case at both 48 h and 72 h of treatment. The IC_50 NO_ for OADP at 48 h (1.09 ± 0.01 μg/mL) and 72 h (0.95 ± 0.01 μg/mL) were significantly lower than for OA (31.28 ± 2.01 μg/mL for 48 h and 42.91 ± 0.27 μg/mL for 72 h) and diclofenac (53.84 ± 2.25 μg/mL for 48 h and 50.50 ± 1.31 μg/mL for 72 h) (Figure 3). Thus, OADP proved to be some 30-fold more anti-inflammatory than OA and about 50-fold more than diclofenac.

### 2.3. Cell Cycle Arrest

**Figure 4:**
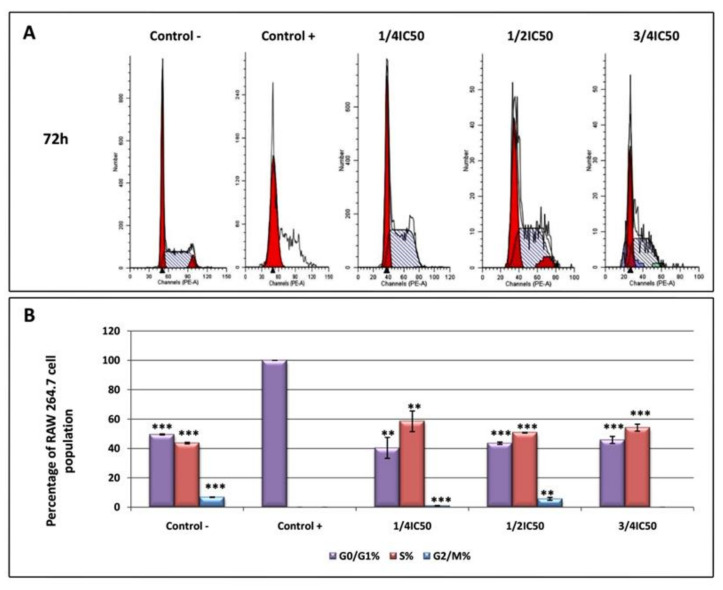
DNA histograms of cell cycle analysis of RAW 264.7 macrophage murine cell, after treatment with OADP for 72h at the ½ IC_50_ and ¾ IC_50_ concentrations (**A**). Percentage of RAW 264.7 macrophage murine cell population in the different phases of the cell cycle (G0/G1, S and G2/M) (**B**). Data represent the mean ± S.D. of at least two independent experiments performed in triplicate. Key: (**) *p* < 0.01 and (***) *p* < 0.001, with respect to LPS-treated control cells (positive control).

Flow cytometry with propidium iodide (PI) staining was used to evaluate the DNA ploidy and alterations in the cell-cycle profiles since the DNA content is directly proportional to the PI fluorescence, which indicates the percentage of cells in each phase of the cycle (Figure 4). RAW 264.7 cells were treated with LPS for 24 h to induce the inflammatory process, after which the cells were treated with OADP for 72 h at the corresponding sub-cytotoxic concentrations. A significant growth arrest occurred during the G0/G1 phase in the positive control (100%) compared to the negative control (49.6%). A DNA histogram analysis revealed that OADP was capable of reversing LPS-induced cell-cycle arrest in the G0/G1 phase. When the cells were treated with OADP at the corresponding sub-cytotoxic concentrations (¼ IC_50_, ½ IC_50_, and ¾ IC_50_), the percentage of cell-cycle arrest was significantly reduced in the G0/G1 phase by up to 40.4%, 43.6%, and 45.9%, respectively. This decrease in the percentage of cells in the G0/G1 phase was accompanied by a concomitant increase in the percentage of cells in the S phase (58.5% at ¼ IC_50_, 50.7% at ½ IC_50_, and 54.2% at ¾ IC_50_). This recovery in the cell cycle could be a result of the anti-inflammatory effects caused by OADP, which appears to have stimulated cell division. These results show no significant variations for the different sub-cytotoxic concentrations used, indicating that OADP has anti-inflammatory effects at all the concentrations tested. The changes were not significant in the G2/M phase of the cell cycle.

### 2.4. OADP-Induced Cell-Morphology Changes on LPS-Stimulated RAW 264.7 Cells

**Figure 5:**
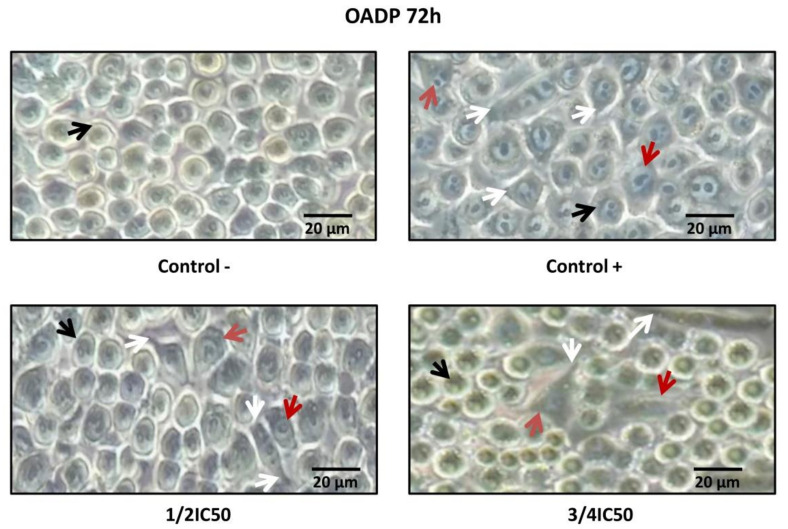
Morphological transformation in RAW 264.7 cells stimulated with LPS and OADP treatment for 72 h at the ½ IC_50_ and ¾ IC_50_ concentrations. The black arrows indicate Raw 264.7 cells in monocyte state. The red arrows indicate Raw 264.7 cells in macrophage state. The white arrows indicate cytoplasmic projections.

The cell morphology of RAW 264.7 cells was analyzed in the presence as well as the absence of LPS and OADP. Treatment of LPS-stimulated RAW 264.7 cells with OADP for 72 h at the sub-cytotoxic ¼ IC_50_ and ¾ IC_50_ concentrations led to morphological changes. In general, untreated cells were smooth and round, whereas LPS-stimulated RAW 264.7 cells had changed to an irregular and rough shape with dendritic formations. Dendritic morphology was characterized by multiple prominent cytoplasmic projections. Co-treatment of LPS with OADP decreased the level of dendritic formation, showing a shape more similar to that of untreated cells, in a dose-dependent manner. The cells were visualized under optical microscopy (Figure 5).

### 2.5. OADP-Induced Inhibition of the Expression of Pro-Inflammatory Proteins TNF-α and IL-1β on LPS-Stimulated RAW 264.7 Cells

**Figure 6:**
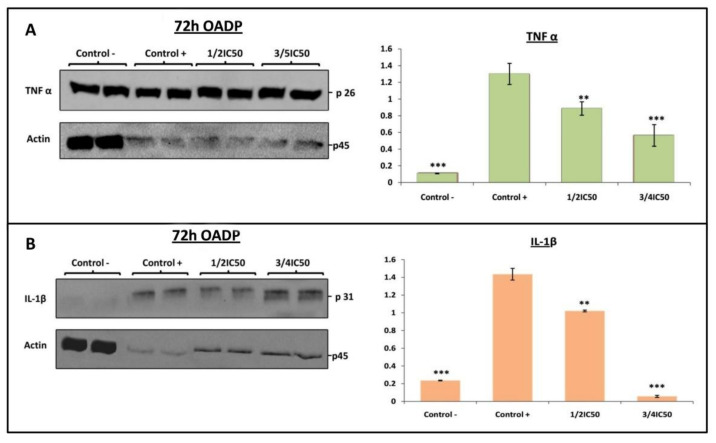
Western blot of the levels of TNF-α (**A**) (p26) and IL-1β (**B**) (p31) proteins on LPS-stimulated Raw 264.7 cells, after treatment with OADP for 72h at the ½ IC_50_ and ¾ IC_50_ concentrations. Protein expression levels are expressed as arbitrary intensity units for each band compared to arbitrary intensity units for actin (p45). Variations in the relative percentages of TNF-α and IL-1β expression for each concentration are also shown. The values represent means ± S.D. of at least two separate experiments. Key: (**) *p* < 0.01 and (***) *p* < 0.001, with respect to LPS-treated control cells (positive control).

The activation of pro-inflammatory cytokines is one of the central processes that occur during the induction of the inflammatory response. TNF-α and IL-1β are potent pro-inflammatory cytokines capable of recruiting immune cells and triggering inflammation. TNF-α is a pro-inflammatory cytokine produced by various immunocompetent cells, including macrophages, neutrophils, Th1/Th2 cells, and dendritic cells. IL-1β is a pro-inflammatory cytokine produced by B cells, endothelial cells, and fibroblast cells. However, its release is linked to an acute and chronic inflammatory process, inducing the acute-phase reaction and favoring prostaglandin synthesis. The results of the Griess test indicated that the strongest anti-inflammatory effect occurred after 72 h of incubation with OADP at the ¾ IC_50_ concentration, accompanied by a weak anti-inflammatory effect at the ½ IC_50_ and ¼ IC_50_ concentrations. Therefore, using Western blot analysis, we examined the expression of the pro-inflammatory cytokines TNF-α and IL-1β after 72 h of incubation with OADP at ¾ IC_50_ and ½ IC_50_ concentrations. In this case, we found that the expression of TNF-α and IL-1β in RAW 264.7 cells increased significantly in the positive control (12-fold with respect to the TNF-α expression and 6-fold with respect to the IL-1β expression, compared to the negative control). Treatment with OADP prompted a concentration-dependent decline in the expression of these proteins. Thus, OADP significantly reduced the expression of TNF-α in RAW 264.7 cells (32% at ½ IC_50_ and 57% at ¾ IC_50_) compared to the positive control (Figure 6A). Furthermore, the level of IL-1β lowered in RAW 264.7 cells (29% at ½ IC_50_ and 96% at ¾ IC_50_) compared to the positive control (Figure 6B). Therefore, OADP proved capable of suppressing the synthesis and release of these cytokines, becoming a possible candidate for the development of new anti-inflammatory drugs.

### 2.6. OADP-Induced Inhibition of COX-2, iNOS, and p-IκBα Protein Expression on LPS-Stimulated RAW 264.7 Cells

The uncontrolled inflammatory process can lead to tissue damage and various inflammatory diseases, such as autoimmune disorders, cardiovascular diseases or tumorigenesis. It is well known that stress and inflammatory cytokines induce phosphorylation and stimulation of active NF-κB, protein involved in the activation of the cell-proliferation process. Furthermore, NF-κB is the main transcription factor regulating the production of inflammatory proteins such as iNOS and COX-2, which are activated by cytokines TNF-α and IL-1β during immune responses.

**Figure 7:**
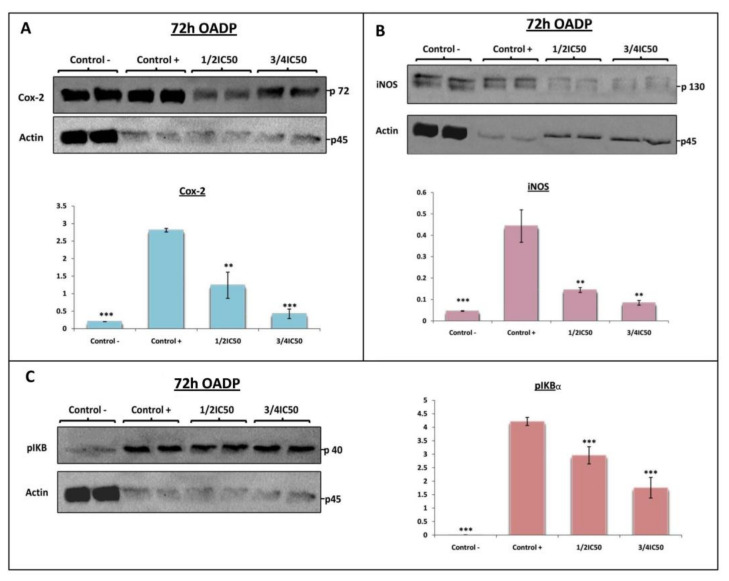
Western blot of the levels of proteins COX-2 (**A**) (p72), iNOS (**B**) (p130), and p-IκBα (p40) (**C**) on LPS-stimulated RAW 264.7 cells, after treatment with OADP for 72h at the ½ IC_50_ and ¾ IC_50_ concentrations. Protein expression levels are expressed as arbitrary intensity units for each band compared to arbitrary intensity units for actin (p45). Variations in the relative percentages of TNF-α and IL-1β expression for each concentration are also shown. The values represent means ± S.D. of at least two separate experiments. Key: (**) *p* < 0.01 and (***) *p* < 0.001, with respect to LPS-treated control cells (positive control).

In this context, the levels of two main inflammatory mediators such as COX-2 and iNOS were examined by Western blot analysis at 72 h after treatment with OADP (¾ IC_50_ and ½ IC_50_ concentrations) in order to determine the anti-inflammatory effect caused in RAW 264.7 cells. The results indicate that in non-stimulated RAW 264.7 cells (negative control) iNOS and COX-2 expression was very low, whereas in LPS-stimulated cells (positive control) the protein levels rose markedly (14-fold with respect to iNOS expression and 10-fold with respect to COX-2 expression, compared to the negative control). As in the previous case, the treatment with OADP prompted a concentration-dependent decrease in the expression of these proteins. Therefore, OADP substantially reversed the high level of expression of COX-2 (56% at ½ IC_50_ and 85% at ¾ IC_50_) and iNOS (67% at ½ IC_50_ and 81% at ¾ IC_50_), induced by LPS treatment in a concentration-dependent manner, compared to the positive control (Figure 7A,B).

The IκBα protein (NF-κB inhibitory protein alpha) inhibited NF-κB, is inactivated and degraded by phosphorylation, resulting in p-IκBα. To further explore the anti-inflammatory role of OADP in LPS-stimulated Raw 264.7 cells, we used Western blot analysis to evaluate the expression level of the p-IκBα protein. This level rose some 480-fold more in LPS-stimulated Raw 264.7 cells than in the negative control (Figure 7C). In addition, OADP significantly reduced the expression level of the p-IκBα protein compared to the positive control. After treatment with LPS for 72 h, the expression levels of the protein p-IκBα clearly increased, but a co-treatment with LPS and OADP at sub-cytotoxic concentrations significantly attenuated this expression in a dose-dependent manner (30% at ½ IC_50_ and 58% at ¾ IC_50_), compared to the positive control (Figure 7C). These results demonstrate that OADP clearly inhibited the expression of the p-IκBα protein in LPS-stimulated RAW 264.7 cells.

### 2.7. OADP Inhibition of TPA-Induced Inflammation in Mouse Ear

**Figure 8:**
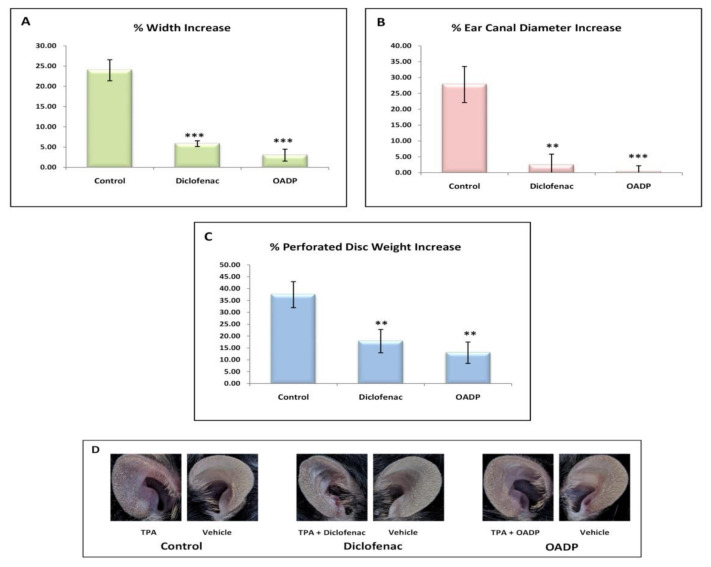
Increases in the percentage of the different morphological parameters: Width (**A**), diameter of the external auditory canal (**B**), and weight of the 6mm disc practiced (**C**). These increases have been calculated from the differences between the left and right ears in the different groups of mice (Control, Diclofenac, OADP) (**D**). The mean ± S.E.M. is shown for each of these parameters (*n* = 4).

The results showed the clear induction of edema in response to treatment with TPA. Induction proved noticeable in all morphological measurements made, with an increase of approximately 25% to 40%, when comparing the right ears with the left, in each of the parameters measured (Figure 8). Thus, the width increased by 25% (23.97 ± 2.6%), the diameter of the external auditory canal by 30% (27.81 ± 5.8%), and the weight of the 6-mm disc (removed from the ear as a sample) by 40% (37.42 ± 5.6%). This effect was also appreciable in the phenotype presented by the right ear in the control mice (Figure 8D).

The TPA-induced edema was significantly inhibited in OADP-treated mice, with virtually no changes between the right and left ear. The width increase (2.99 ± 1.48%) was 22% less than in the control. No differences were detected in the diameter change of the external auditory canal on comparing the two ears (0.18 ± 6.10%), this being some 30% less than in the control. The weight of the 6-mm disc removed from the ear increase (12.90 ± 5.47%), being 24.5% less than in the control.

The results for the treatment with diclofenac were similar to those found with OADP, although higher values were registered for the increases in the different parameters evaluated: 5.83 ± 0.71% in the width, 2.34 ± 3.5% in the diameter of the external auditory canal, and 17.84 ± 4.90% in the weight of the 6-mm disc removed from the ear.

### 2.8. OADP-Induced Decreases in TPA-Induced Mouse Ear Injury

**Figure 9:**
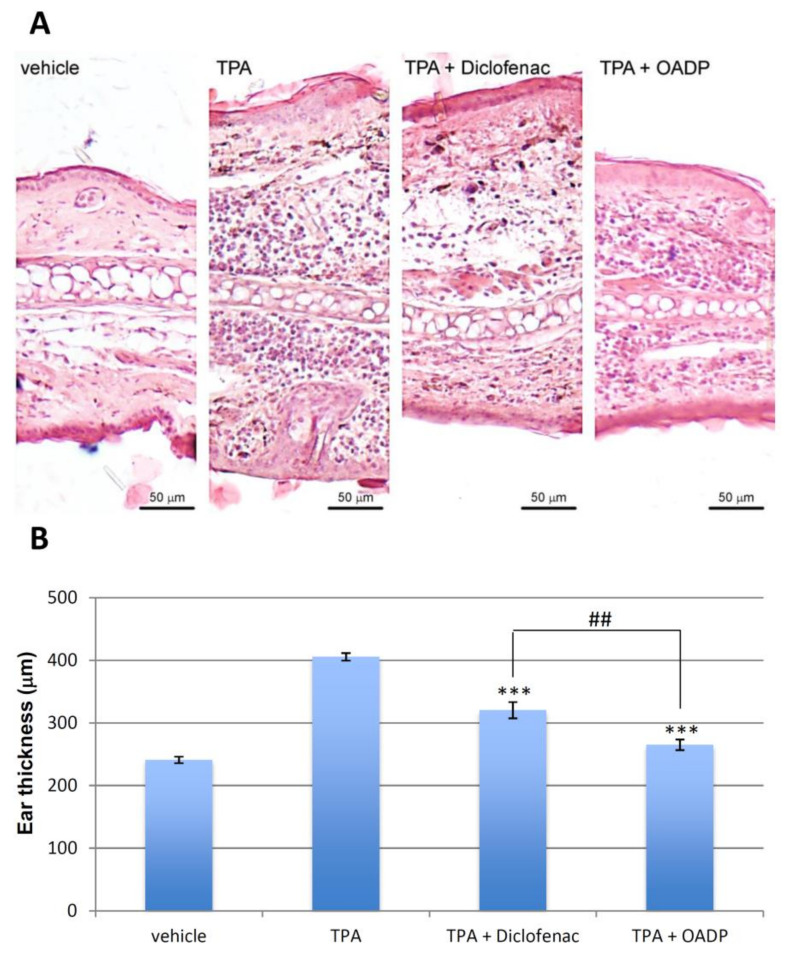
Representative photomicrographs. (**A**) The images show a cross section of the ears treated with vehicle (acetone), TPA alone, TPA + Diclofenac, TPA + OADP, at a 125x magnification. Scale bars = 50 µm. (**B**) The graph represents the mean thickness of the ear of each treatment, expressed as mean ± S.E.M. (***) *p* < 0.001 compared to TPA treated ears. (^##^) *p* < 0.01 compared to the ears treated with TPA + Diclofenac.

The main effect of OADP was studied in mouse edema biopsies in response to TPA treatment. In the control group, the right ear (treated with TPA) compared to the control left ear displayed intense tissue destruction and edema, with infiltrates of inflammatory polymorphonuclear leukocytes, mainly neutrophils, and hyperplasia as well as hypertrophy of the dermis and epidermis (vehicle, Figure 9A). However, pre-treatment with diclofenac and OADP significantly lowered the level of infiltration of edematous and inflammatory cells, compared to the control treated with TPA (Figure 9A). Furthermore, diclofenac and OADP reduced these impairments, markedly reducing ear thickness (by 21% and 35%, respectively) compared to the TPA-treated control (Figure 9B). Topical pre-treatment with OADP alleviated the lesion more effectively than did diclofenac, as it notably depressed the development of erythema (Figure 9A,B).

### 2.9. OADP-Induced Decreases in IL-6 Released by TPA in the Mouse Ear

**Figure 10:**
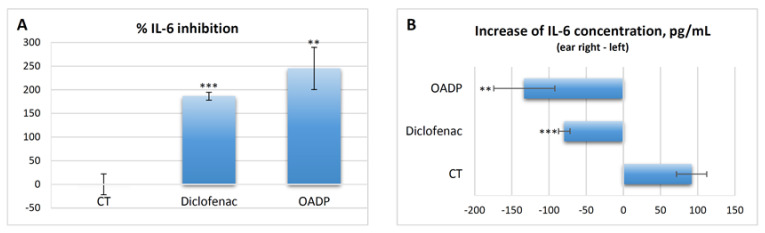
IL-6 inhibition percentage, calculated from the differences between the left and right ears in the different groups of mice (**A**). Increases in IL-6 concentration (pg/mL) calculated from the differences between the left and right ears in the different groups of mice (control, diclofenac and OADP) (**B**). The mean ± S.E.M. is shown for each of these parameters (*n* = 4). Key: (**) *p* < 0.01 and (***) *p* < 0.001, with respect to the control group of mice.

Comparisons of the IL-6 concentrations between the right and left ear within each group of animals revealed a clear induction of inflammation in the control group, with an increase in IL-6 concentration of 91.84 ± 20.23 pg/mL in the right ear compared to the left (Figure 10A). In the rest of the groups treated with the different inhibitors, including diclofenac, no increase was detected in the right ear compared to the left, and the interleukin-6 concentration clearly decreased in this comparison (Figure 10B). Diclofenac lowered the IL-6 concentration by 180% (186.41 ± 8.37%), while OADP caused a fall of almost 250%, representing 60% stronger inhibition (Figure 10). Thus, regarding the production of IL-6, we conclude that OADP is clearly more efficient in vivo than is diclofenac.

## 3. Discussion

Inflammation is a common physiological response that when acute can be protective but when chronic can cause a wide variety of diseases, including cancer [26]. Therefore, inflammation becomes fundamental in tumor development, stirring intense interest in developing new anti-inflammatory agents that have significant anticancer properties [27]. Natural products, often used as an alternative to synthetic drugs in these types of afflictions, include oleanolic acid and its derivatives, which offer potential therapeutic effects in chronic diseases [28].

Previous results have shown that OADP can induce apoptosis at very low concentrations in different cancer cell lines [16,17]. In the present study, we examine the potential of OADP as an anti-inflammatory agent and investigate the underlying molecular mechanism for this effect in LPS-stimulated RAW 264.7 macrophage cells. Furthermore, we performed in vivo tests on a TPA-induced acute mouse ear edema model to evaluate the suppressive effect of OADP on pro-inflammatory mediators.

During the inflammation process, various types of leukocytes, lymphocytes, and other inflammatory cells are activated. Macrophages prove crucial in various inflammatory diseases by inducing the expression of pro-inflammatory mediators [29]. LPS is a strong inducer of monocytes to macrophages, stimulating the production of pro-inflammatory mediators [30]. Macrophage stimulation is represented by expanded cell size and extension of the cytoplasm. Other changes in stimulated macrophages serve to amplify the immune response [31]. OADP cytotoxicity was determined in murine monocyte/macrophage RAW 264.7 cells in order to establish sub-cytotoxic concentrations of this compound. The viability of these cells was tested at various OADP concentrations, using an MTT assay, which gave an IC_50_ concentration of 1.73 µg/mL.

The primary pro-inflammatory mediator for acute or chronic inflammation is NO. In general, NO inhibitors provide excellent opportunities to design new therapeutic methods for inflammatory diseases [32]. In the present work, the inhibitory activity of OADP against NO release in LPS-stimulated RAW 264.7 cells is studied. OADP improved the NO inhibitory activity compared to the positive control (Figure 2). At 72 h of incubation, OADP resulted in 75%, 25%, and 21% inhibition of NO, for concentrations of ¾ IC_50_, ½ IC_50_ and ¼ IC_50_, respectively, with respect to the positive control. To compile more insight on this anti-inflammatory effect, we calculated the effective doses at 50% NO inhibition (IC_50 NO_) after 48 h and 72 h of treatment with OADP and found that the IC_50 NO_ for OADP at 48 h were significantly lower than for OA and diclofenac, being 30-fold more anti-inflammatory than OA and about 50-fold more than diclofenac (Figure 3). The flow-cytometry results revealed the anti-inflammatory effect of OADP in RAW 264.7 cells at the corresponding sub-cytotoxic concentrations tested. Furthermore, OADP exhibited anti-inflammatory activity at 72 h of treatment by reversing the cell-cycle arrest induced by LPS (Figure 4). Morphological changes of RAW 264.7 cells were viewed under a light microscope (×400). Untreated cells (negative control) were circular but became irregular in shape with multiple prominent cytoplasmic projections and dendritic formations, after stimulation with LPS (positive control). After 72 h of treatment with OADP, at different sub-cytotoxic concentrations, the degree of cell propagation and dendritic formation diminished markedly, in a dose-dependent manner (Figure 5).

The NF-κB and MAPK pathways are essential in regulating the production of inflammatory cytokines in activated macrophages such as TNF-α, IL-1β, and IL-6. TNF-α participates in various processes such as cell survival, apoptosis, and necrosis, by stimulating TNF receptors and related pathways, such as NF-κB and MAPKs [33]. The LPS-induced pattern of macrophage activation leads to high production of inflammatory mediators, such as TNF-α, IL-1β, and IL-6. This model is widely used to detect anti-inflammatory drugs [34].

Triterpenoids and their derivatives have an effective inflammatory effect both in vitro and in vivo. Inhibition of MAPKs pathways in the anti-inflammatory actions of several triterpenoids has been observed. For example, ursolic acid (UA) inhibited mitogen-induced phosphorylation of ERK and JNK and prevented the activation of immunomodulatory transcription factors such as NF-κB, NF-AT, and AP-1 in T and B lymphocytes [35]. OA decreased the levels of TLR4 and NF-κB and MAPKs in the mouse model with salmonella-induced intestinal inflammation [36]. Indole OA derivatives, inhibited the expression of proteins of p-p38, p-JNK, p-ERK, p-NF-κB p-Akt, iNOS and COX-2, and enhanced the expression of Nrf2 in LPS-activated BV2 cells [37].

Lupeol has been found to impede the expression of pro-inflammatory cytokines such as TNFα and IL-β in LPS-stimulated macrophages [38]. Additional studies have shown that maslinic acid exerts an anti-inflammatory effect by inhibiting the production of NO induced by oxygen and glucose deprivation, TNF-α suppressing the expression of COX-2 and iNOS at the levels of protein and mRNA [39]. Maslinic acid reportedly exerts its anticancer effects on HT29 colon-cancer cells through a JNK-p53 dependent mechanism [40]. In a chemoprevention of tumorigenesis in ApcMin/+ mice, maslinic acid has been found to inhibit molecular pathways of inflammation and cell survival [41]. On the other hand, OA reportedly prevented colitis by inhibiting Th17 cells and the down-regulation of the expression of interleukin IL-1β, NF-κB, MAPK, and RORγt in the colon [42]. CDDO-Me, a semi-synthetic derivative of OA has been found to induce downregulation of the expression of F4/80, CD11c, COX-2, IL-6, KI67, NF-B, and TNF-α [43].

In this context, to investigate whether the anti-inflammatory effects after 72 h of OADP treatment were associated with the activation of TNFα and IL-1β, we performed a Western blot analysis and found that LPS markedly increased the production of TNF -α and IL-1β in inflammatory responses in RAW 264.7 cells. Meanwhile, OADP reversed the LPS-induced production of TNF-α and IL-1β after 72 h of treatment. In addition, a notable decrease in TNF-α expression was recorded in RAW 264.7 cells (32% at ½ IC_50_ and 57% at ¾ IC_50_, Figure 6A), accompanied by a significant fall in the level of IL-1β (29% at ½ IC_50_ and 96% to ¾ IC_50_, Figure 6B). These results imply that OADP has a protective effect on LPS-induced inflammation in RAW 264.7 cells. Thus, OADP is capable of suppressing the release of TNF-α and appears to be an excellent choice for use as a model in developing efficient anti-inflammatory drugs.

The two major enzymes iNOS and COX-2 induce the production of two crucial inflammatory mediators, NO and PGE2, respectively [44]. Furthermore, increased iNOS expression prompts high levels of NO and has been associated with various chronic inflammatory disorders [45]. Prostaglandin E2 (PGE2), biosynthesized by COX-2 from arachidonic acid, is a major inflammatory mediator, increasing local blood flow, pain sensitization, and edema. The inhibition or down-regulation of COX-2 expression blocks PGE2 synthesis and inhibits inflammation [46].

In the present work, the expression of COX-2 and iNOS was evaluated by Western blot analysis after 72 h of OADP treatment in LPS-activated RAW 264.7 cells. OADP exerted a gradual anti-inflammatory effect in a concentration-dependent manner from ½ IC_50_ to ¾ IC_50_, this effect being linked to the decline in the expression of COX-2 (56% at ½ IC_50_ and 85% at ¾ IC_50_) and iNOS (67% at ½ IC_50_ and 81% at ¾ IC_50_) (Figure 7A,B). Furthermore, the level of p-IκBα was studied at the corresponding sub-cytotoxic concentrations, revealing that phosphorylation of IκBα was significantly inhibited after treatment with OADP in a concentration-dependent manner (30% at ½ IC_50_ and 58% at ¾ IC_50_), relative to only the LPS-treated RAW 264.7 cells (positive control, Figure 7C).

The TPA-induced mouse ear edema model is widely used to study the anti-inflammatory activity of new compounds [47] Pentacyclic triterpene α-amyrin inhibited TPA-induced activation of PKCα, ERK, and p38 MAPK, thereby preventing IκBα degradation and p65/RelA phosphorylation [48].

The anti-inflammatory effect of diclofenac and OADP was evaluated in mice having acute ear edema induced by TPA, and the treatment was found to be remarkably effective after 6 h of treatment. This anti-inflammatory effect was analyzed as a percentage of edema suppression in the treated groups, in contrast to the control group (vehicle), determining a series of morphological measurements. TPA caused a strong inflammatory process, characterized by a significant increase in the percentage of length, width, thickness, diameter of the external auditory canal and weight (Figure 8). However, treatment with diclofenac and OADP showed a significant anti-inflammatory effect by reducing these morphological measurements. These results were confirmed by a histopathological analysis (Figure 9), where OADP reduced edema and leukocyte infiltration better than did diclofenac. The production of IL-6 was also evaluated. Treatment with OADP stemmed IL-6 production more effectively than did the diclofenac treatment, resulting in a 250% inhibition in cytokine production, this being 60% greater than with diclofenac (Figure 10).

In this sense, other triterpenoids such as Lupeol, have been effective against TPA-induced inflammation in acute ear edema mouse model, which also decreased myeloperoxidase levels causing a reduction in cellular infiltration in inflamed tissues, significantly inhibiting PGE2 levels [38]. Lupeol reduced cellularity and eosinophil levels in broncho-alveolar fluid [49]. This compound has also been shown effective against inflammation in the arthritis mouse model [50]. Another pentacyclic triterpenoid such as ursolic acid (UA) has shown anti-inflammatory activity in activated T cells, B cells and macrophages, and against graft-versus-host disease in vivo mouse model, significantly reducing serum levels of pro-inflammatory cytokines IL-6 and IFN-γ [35]. Indole oleanolic acid derivatives inhibited the expression of pro-inflammatory cytokines (TNF-α, IL-6, IL-12 and IL-1β) and increased the expression of anti-inflammatory cytokine IL-10 [37].

**Figure 11:**
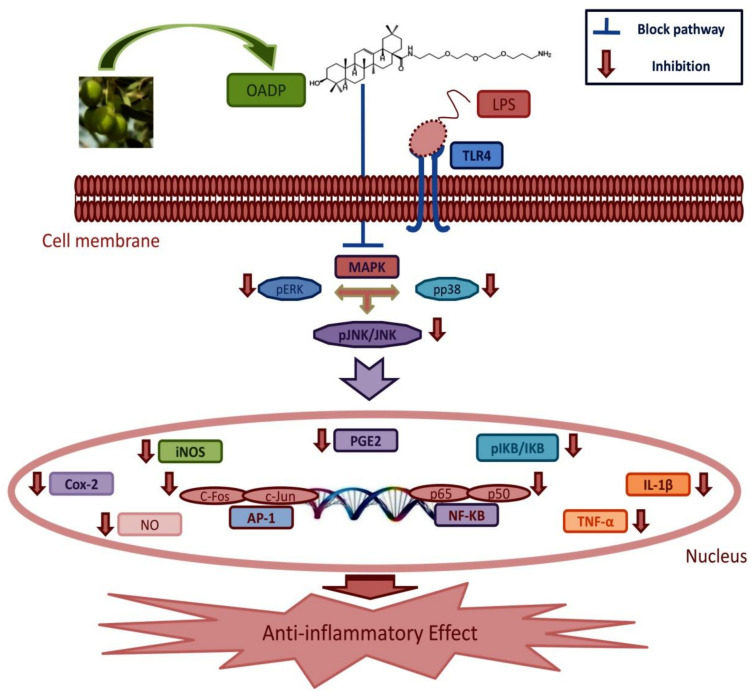
Mechanism of action underlying the anti-inflammatory effect induced by OADP in LPS-stimulated RAW264.7 cells, after 72 h of treatment, at the ½ IC50 and ¾ IC50 concentrations. This process triggers the inhibition of phosphorylation of IκBα, the inhibition of the pro-inflammatory cytokines TNF-α and IL-1β. Inhibition of the inflammatory mediators COX-2 and iNOS, induced inhibition of NO production.

With the results found and considering the bibliographic data on the anti-inflammatory action of triterpene compounds, the following molecular mechanism was proposed for the anti-inflammatory action of OADP (Figure 11). OADP could inhibit the activation of the TLR4 or TNFR2 receptors, preventing the activation of MAP kinases (ERK, JNK or p38) or inhibiting the phosphorylation of IκBα, which would lead to the inhibition of the activation of pro-inflammatory transcriptors such as NF-κB or the AP1 set (c-Jun, c-Fos, JunB, or JunD). Finally, all this could produce the inhibition of the expression of pro-inflammatory cytokines such as TNFα, IL-1β or IL-6, and the proteins that are expressed in pro-inflammatory processes such as COX-2 or iNOS.

The mouse model with acute ear edema has been chosen due to the low rate of suffering exerted in animals, compared to other tumorigenic inflammation models, in which TPA is used as a tumor promoter. For example, induction of skin carcinoma, in a two stages carcinogenesis mouse model [51], where the tumorigenic process is initiated with 7,12-dimethylbenz[a]-anthracene (DMBA) and the animals are treated for weeks with TPA. Our main objective in this article is to demonstrate the effectiveness of OADP as an anti-inflammatory agent and to study its behavior against inflammation induced by tumor promoters as TPA. OADP has been shown to be a very effective agent against tumor cell proliferation in HepG2 human hepatoma cancer cell line [17], and against inflammatory processes. Due to these results, we defend that OADP can be a good agent against tumorigenic processes. However, it will be necessary to accomplish future trials in inflammation models derived from cancer growth or genetic defects or chronic tissue degeneration/infection, similar to those performed with maslinic acid in intestinal tumorigenesis in ApcMin/+ mice [41]. Further studies will be necessary to establish the anti-inflammatory potential of OADP against chronic inflammation diseases such as arthritis or Parkinson’s. In this sense, it would be interesting to determine the role of OADP to counteract inflammation after neurodegeneration, in microglia murine cell line model stimulated by LPS [52]. Although the anti-tumor and anti-inflammatory potential of OADP is clear, it will be necessary to perform all these studies before carrying out any clinical trials.

## 4. Materials and Methods

### 4.1. Materials

RPMI 1640 W/L-Glutamine, fetal bovine serum (FBS), gentamicin (Biowest, Nuaillé, France), DMSO (Merck Life Science S.L., Madrid, Spain), and 3-(4,5-dimethylthiazol-2-yl)-2,5-diphenyltetrazolium bromide (MTT) (Thermo Fisher Scientific Inc., Ward Hill, MA, USA). IL-1β, tumor necrosis factor alpha TNF-α, p-IκB-α, iNOS, and COX-2 primary antibodies, and anti-rabbit, anti-goat, and actin secondary antibodies were purchased from Santa Cruz Biotechnology (Santa Cruz, CA, USA). Phorbol ester 12-*O*-tetradecanoylphorbol-13-acetate (TPA), culture flasks, and well plates were obtained from VWR International, Ltd. (Radnor, PA, USA).

### 4.2. Test Compounds

Oleanolic acid (OA) was isolated from solid olive oil production wastes, which were extracted successively in a Soxhlet extractor with hexane and EtOAc. Hexane extracts were a mixture from which OA was purified by column chromatography over silica gel followed by elution with CH_2_Cl_2_/acetone mixtures of increasing polarity [12].

A solution of di-tert-butyl dicarbonate (Boc_2_O, 2.75 mmol) in dried CH_2_Cl_2_ (2 mL) was added slowly to a solution of 4,7,10-trioxatridecane-1,13-diamine (H_2_N-PEG-NH_2_, 6.8 mmol) in CH_2_Cl_2_ (20 mL). The reaction mixture was maintained at room temperature for 12 h, and then was diluted with water and extracted three times with CH_2_Cl_2_. After the organic layer was dried with anhydrous Na_2_SO_4_, the solvent was removed under reduced pressure, and thus the H2N-PEG-NH-Boc (85%) was produced [16]. In a flask (20 mL), the compound H2N-PEG-NH-Boc (0.45 mmol) was dissolved in DMF (5 mL) and afterwards OA (2 mmol), HOAt (3 mmol), PyAOP (2 mmol), and DIPEA (8 mmol), were added. The reaction mixture was heated at 100 °C for 12 h, diluted with water and extracted three times with CH_2_Cl_2_. The organic layer was dried with anhydrous Na_2_SO_4_ and the solvent was removed under reduced pressure. Finally, the residue was purified by column chromatography, yielding the OA-diamine-Boc-PEGylated derivative (94%) [16]. After this OA-diamine-Boc-PEGylated derivative (0.3 mmol) was dissolved in THF (20 mL), concentrated HCl (37%, 2 mL) was added. The reaction mixture was maintained at room temperature for 24 h, and then was diluted with water and extracted three times with CH_2_Cl_2_. The organic layer was dried with anhydrous Na_2_SO_4_, and the solvent was removed under reduced pressure. Finally, the residue was purified by column chromatography, yielding OADP (95%) [16].

**Figure 12:**
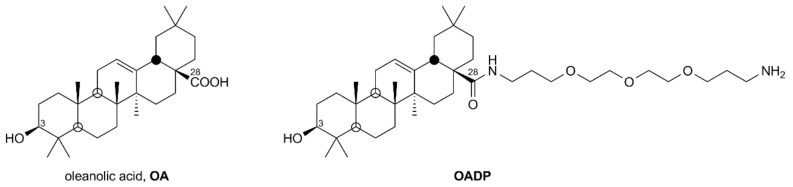
Structures of triterpene compounds **OA** and **OADP**.

Finally, OA and OADP (Figure 12) were dissolved in DMSO at 5 mg/mL and stored at −20 °C. Prior to treatment, the stock solution was diluted in cell culture medium to the appropriate concentration for each experiment.

### 4.3. Cell Culture

The RAW 264.7 monocyte/macrophage cell line (ATCC no. TIB-71) is a murine leukemia virus-induced tumor cell line from mouse Mus musculus. This cell line does not produce detectable retrovirus. The RAW 264.7 cell line was cultured in RPMI1640 medium, supplemented with 2 mM glutamine, 10% heat-inactivated FCS, 0.5 μg/mL of gentamicin, and incubated at 37 °C in a 5% atmosphere of CO_2_ at 95% humidity. Cells were grown to 80–90% confluence in sterile cell-culture flasks. Sub-confluent monolayer cells were used in all experiments. The cell line was provided by the cell bank of the University of Granada, Spain.

### 4.4. Cell-Viability Assay

The OA and OADP assay on RAW 264.7 cells was evaluated using the MTT proliferation assay. Cell viability was evaluated by measuring the absorbance of MTT staining of live cells. For this test, 6 × 10^3^ RAW 264.7 cells were grown in a 96-well plate and subsequently incubated with OA and OADP at different concentrations (0–100 μg/mL). After 72 h of incubation, 100 μL of MTT solution (0.5 mg/mL) in 50% of PBS with 50% of medium was placed in each well. After 1.5 h of incubation, the formazan was resuspended in 100 μL of DMSO. Finally, the relative cell viability, with respect to the untreated control cells, was evaluated by absorbance at 570 nm in an ELISA plate reader (TecanSunrise MR20–301, TECAN, Austria). The experimental data were fitted to a sigmoid function (y = ymax/(x/a)^-b^) by non-linear regression. IC_50_ values were obtained by interpolation. Similar analyses were performed to determine the IC_50 NO_ of NO production (vide infra). All these analyses were performed with the statistical software SigmaPlot (Version 12.5). The values of cell viability were expressed as means ± S.D. of at least two experiments performed in quadruplicate for each concentration.

### 4.5. Determination of the NO Concentration

The nitrite concentration was used as an indicator of NO production. The determination of the nitrite concentration in the culture medium was evaluated according to the Griess reaction. Cells were plated at 6 × 10^4^ cells/well in 24-well cell culture plates and supplemented with 10 μg/mL of LPS. After 24h of plating, cells were incubated for 24, 48, and 72 h with OADP at ¼ IC_50_, ½ IC_50_, and ¾ IC_50_ concentrations. The supernatants were collected at 24 h, 48 h, and 72 h to determine their nitrite concentration and/or stored at −80 °C for later use. The Griess reaction was performed by taking 150 μL of supernatant test sample or the sodium nitrite standard (0–120 μM), mixing with 25 μL of Griess reagent A [0.1% N-(1-naphthyl)ethylenediamine dihydrochloride] and 25 μL of Griess reagent B (1% sulphanilamide in 5% of phosphoric acid) in a 96-well plate. After 15 min of incubation at room temperature, the absorbance at 540 nm was determined in an ELISA plate reader (Tecan Sunrise MR20-301, TECAN, Austria). The absorbance was referred to the nitrite standard curve to determine the nitrite concentration in the supernatant of each experimental sample. The percentage of NO production was determined, assigning 100% to the increase between the negative control (untreated cells) and the positive control (cells treated only with 10 μg/mL of LPS). The values of NO concentration were expressed as means ± S.D. of at least two experiments performed in triplicate for each concentration.

### 4.6. Cell-Cycle Analysis

PI staining flow cytometry constitutes a fast, efficient, and reproducible method for determining relative DNA content. Flow cytometry provides an estimate of alterations in cell-cycle profiles and characteristic changes in DNA levels of cell-cycle arrest and cell differentiation. The number of cells at each stage of the cell cycle is determined by fluorescence-associated cell sorting (FACS) at 488 nm on an Epics XL flow cytometer (Coulter Corporation, Hialeah, FL, USA). For this test, 12 × 10^4^ LPS-stimulated murine macrophage/monocyte RAW 264.7 cells were placed in 24-well plates with 1.5 mL of medium and incubated with OADP for 24 h at ¼ IC_50_, ½ IC_50_, and ¾ IC_50_ concentrations. The positive control consisted of cells treated only by LPS stimulation, while the negative control comprised cells not treated with LPS. The treated cells were LPS-stimulated RAW 264.7 cells and cells treated with the compounds under study. Subsequently, the cells were washed twice with PBS, harvested by tripsinization, and then resuspended in TBS 1X (10 Mm Tris, 150 Mm NaCl), after which Vindelov Buffer (100 mM Tris, 100 Mm NaCl, 10 mg/mL RNAse, and 1 mg/mL PI) at pH 8.0 was added. The samples were placed on ice for 15 min. Immediately before FACS analysis, the cells were stained with 20 μL of 1 mg/mL PI solution. The data were analyzed with the Multicycle software to determine the percentage of cells in each phase of the cell cycle (G0/G1, S, and G2/M). The values of cell percentage were expressed as means ± S.D. of at least two experiments performed in triplicate for each concentration.

### 4.7. Western Blot Analysis

RAW 264.7 cells (12 × 10^4^) were treated with OADP at the sub-cytotoxic concentrations cited above, for 72 h. The positive control consisted of cells treated only with LPS stimulation while the negative control was made up of untreated cells without LPS. After the treatments, cells were washed twice with PBS and resuspended in lysis buffer (20 mM Tris/acetate, pH 7.5, 1 mM EDTA, 1 mM EGTA, 1% Triton X-100, 1 mM orthovanadate, 270 mM sucrose, 1 mM sodium glycerophosphate, 5 mM sodium fluoride, 1 mM sodium pyrophosphate, 5 mM β-mercaptoethanol, 1 mM benzamidine, 35 µg/mL PMSF, and 5 µg/mL leupeptin). Samples were homogenized, ultra-sonicated, and incubated on ice for 20 min, before centrifuging at 12,000× *g*, for 15 min. Supernatants were used to calculate the protein concentration, which was evaluated by the Bradford method. For Western blot analysis, a sample of 25 to 50 µg of total protein was used. The proteins were separated on 15% sodium dodecyl sulfate (SDS)-polyacrylamide gel and transferred to polyvinylidene difluoride membranes. The membranes were blocked by incubation for 1 h in TBS buffer containing 0.1% Tween and 5% milk powder at room temperature and washed with TBS buffer containing 0.1% Tween. The membranes were blotted overnight at 4 °C, with primary antibodies: rabbit polyclonal interleukin IL-1β (1/200 dilution), goat polyclonal TNFα (1/100 dilution), goat polyclonal p-IκBα (1/200 dilution), rabbit polyclonal NOS2 (1/200 dilution), and goat polyclonal COX-2 (1/500 dilution). The blots were then washed 3 times with TBS-0.1% Tween, and developed with secondary antibodies bound to peroxidase, for 1 h at room temperature (1/3000 dilution). The blots were then washed 3 times with TBS-0.1% Tween and once with TBS. Afterwards, all blots were revealed using the ChemiDoc XRS Image System (Bio-Rad Laboratories, Hercules, CA, USA). The protein bands were quantified using the Multi-Gauge program (Fuji Film Europe, TK Tiburg, Holland). The data of protein expression were expressed as means ± S.D. of at least three experiments performed in duplicate for each concentration.

### 4.8. Animals

Male BL/6J mice, 8 weeks old and weighing 21–28 g, were purchased from the Animal Experimentation Service of the Center for Scientific Instrumentation of the University of Granada (Spain). These mice were housed for 7 days at 22 °C with 70% humidity, a 12 h light/dark cycle and were fed a standard diet and water was provided ad libitum before experimentation. The experiment was conducted following the guidelines issued by the Animal Care Committee and accepted by the Institutional Ethics Committee of the University of Granada.

### 4.9. TPA-Induced Acute Ear Edema

Edema was induced by topical administration of TPA in acetone, specifically 2.5 µg/ear. In groups of 8 individuals, the mice were treated on both surfaces of the right ear using a 20-μL solution of acetone containing OADP (0.5 mg/ear), while the reference group was treated similarly using diclofenac (0.5 mg/ear) as a reference drug, the control group was not treated with any anti-inflammatory compound. Simultaneously, the TPA was placed on both sides of the right ear at 0, 6, and 18 h. At 6 h after the end of the last treatment, the animals underwent cervical dislocation. The samples were then stored at −80 °C. The left ear was treated with acetone only, as a control. A 6-mm diameter disc was removed from treated as well as untreated ears and weighed separately on an analytical balance. The degree of edema was determined by the weight increase of the right ear over the left. The anti-inflammatory effect was evaluated as a percentage of the suppression of edema in the treated groups in contrast to the control group. A series of morphological measurements were taken in these tissues, such as length, width, thickness, diameter of the external auditory canal, and weight, as detailed below. Length measurements were made with a precision digital caliper.

### 4.10. Histology Study of Acute Ear Edema

The ears of the mice were treated as described above. Subsequently, they were cut and fixed in 4% PFA for 24 h. After three washes in PBS, they were embedded in paraffin and cut into sections on a microtome (Zeiss). The sections were stained with hematoxylin and eosin. Images were taken using an Axiophot microscope (Zeiss) with a magnification of 125×. For its quantification, the thickness of the ear was measured using ImageJ imaging software (NIH) and expressed as mean ± standard error of the mean (S.E.M.). Student’s t-test was applied for the statistical analysis, and a value of *p* < 0.05 was considered statistically significant.

### 4.11. Interleukin-6 Release

After the activation of the inflammation process, key proteins that mediate this process are induced. These proteins are cytokines and proteins responsible for the arachidonic acid cascade. The concentration levels of IL-6 were analyzed as markers of inflammation in the aforementioned different groups of mice.

The tissue samples were washed with cold PBS and then homogenized at 10% tissue concentration in homogenization buffer (PBS, 0.1 M PMSF, 0.5% BSA, 10 mM EDTA, leupeptin 5 μg/mL). Two freeze-thaw cycles were performed to break down the cell membranes. The homogenates were centrifuged at 5000× *g* for 5 min. Finally, the supernatants were removed and immediately analyzed for the cytokine concentration. The IL-6 concentration was determined by the ELISA technique, using a specific kit (E0079m, EIAab Science Co., Wuhan, China). The percentage of IL-6 inhibition was calculated according to:

(1) $$\%\;IL_{6}\;inhibition=\;\;\left\{1-\;\frac{{\left({\left[IL_{6}\right]}_{ear_{right}}-{\left[IL_{6}\right]}_{ear_{left}}\right)}_{sample}}{{\left({\left[IL_{6}\right]}_{ear_{right}}-{\left[IL_{6}\right]}_{ear_{left}}\right)}_{control}}\right\}\times 100$$

### 4.12. Statistical Analysis

The data are represented as the mean ± standard deviation (S.D.). For each assay, the Student’s t-test was used for statistical comparisons with control cells. A limit of *p* ≤ 0.05 was used to assess significant differences: *p* < 0.05 (*), *p* < 0.01 (**) and *p* < 0.001 (***). The data shown are representative of at least two independent experiments performed in triplicate.

## 5. Conclusions

In the present study, we demonstrate that OADP has a powerful anti-inflammatory effect in vitro and in vivo in the models evaluated. Moreover, we deduced the underlying molecular mechanism for the anti-inflammatory effect of OADP on LPS-stimulated RAW 264.7 cells, after 72 h of treatment, at concentrations of ½ IC_50_ and ¾ IC_50_. That is, OADP weakened the expression of pro-inflammatory cytokines such as TNFα and IL-β in LPS-stimulated macrophages. This in turn decreased inflammatory proteins such as iNOS and COX-2 and inhibited NO production, a crucial inflammatory mediator. Furthermore, IκBα phosphorylation was significantly suppressed after 72 h of OADP treatment.

Among the most outstanding results of this study is the potent anti-inflammatory effect of OADP in LPS-stimulated RAW 264.7 cells and in mice with acute ear edema. The results demonstrate that OADP inhibits NO production and reverses the differentiation processes in LPS-stimulated RAW 264.7 cells. Furthermore, OADP impedes the expression of TNF-α, IL-1β, iNOS, and COX-2, as well as blocking the production of p-IκBα in these macrophage cells. Based on these results, the following mechanism is suggested for the anti-inflammatory effect of OADP in these macrophage cells. Firstly, OADP diminishes the expression of TNF-α and IL-1β and decreases iNOS as well as COX-2, which in turn subsequently hinders p-IκBα and NO production (Figure 11). Thus, these results suggest the possible pharmacological use of OADP as a potent and effective anti-inflammatory agent in acute and chronic inflammatory diseases.

## Data Availability

Not applicable.

## Abbreviations

AP-1	Activatior protein 1
COX-2	Cyclooxygenase-2
IC_50_	Total concentration to obtain a 50% cell growth inhibition
IC_50 NO_	Doses that result in 50% NO inhibition
IL	Interleukin
INF-γ	Interferon-γ
iNOS	Inducible nitric oxide synthase
PI	Propidium iodide
IκBα	NF-κB inhibitory protein alpha
JAK	Janus-activated kinase
LPS	Bacterial lipopolysaccharide
MAPK	Mitogen-activated protein kinase
NF-κB	Nuclear factor kappa B
NO	Nitric oxide
NSAID	Nonsteroidal anti-inflammatory drug
OADP	Diamine-PEGylated derivative of oleanolic acid
PI3K/AKT	Phosphatidylinositol-3-kinase
STAT	Signal transducer activator of transcription
TLR	Toll-like receptor
TNFα	Tumor necrosis factor α
TPA	12-*O*-tetra-decanocanoylphorbol-13-acetate
